# Supplementary material for: The Molecular, Morphological and Genetic Characterization of Glyphosate Resistance in Conyza bonariensis from South Africa
Source: Plants (Basel). 2022 Oct 24;11(21):2830. doi: 10.3390/plants11212830 (PMC9654701; doi:10.3390/plants11212830)
Supplement: Supplementary file 1 [file plants-11-02830-s001.zip › plants-1915683-supplementary.pdf]

SUPPLEMENTARY MATERIALS

Supplementary Figures

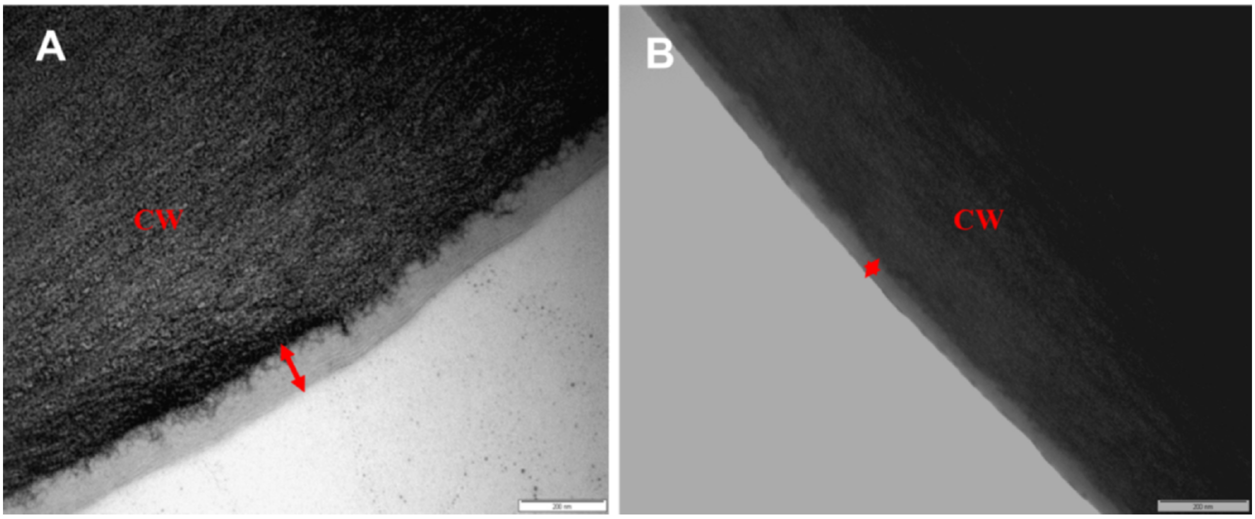

Figure S1. Transmission electron micrographs of outer epidermis cell wall of *C. bonariensis* showing cuticular membrane indicated with red double-sided arrows and cell wall (cw). Image 'A' represents cuticular membrane of resistant biotype (Swellendam - WP 22) while 'B' susceptible biotype (George - WP 19). Scale bar represents 200 nm in each image.

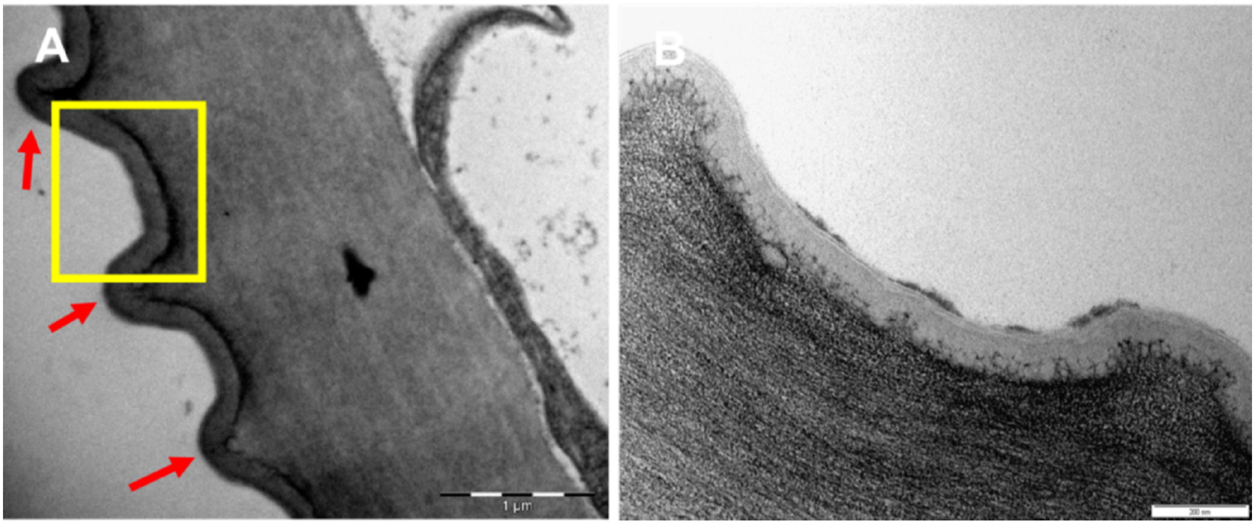

Figure S2. Transmission electron micrographs of outer epidermis cell wall of *C. bonariensis* showing cuticular undulations at lower magnification image ‘A’ (indicated by red arrows) and at higher magnification (Image B) of part indicated by yellow square in image ‘A’. (Scale bar in A = 1 µm and 200 nm in B).

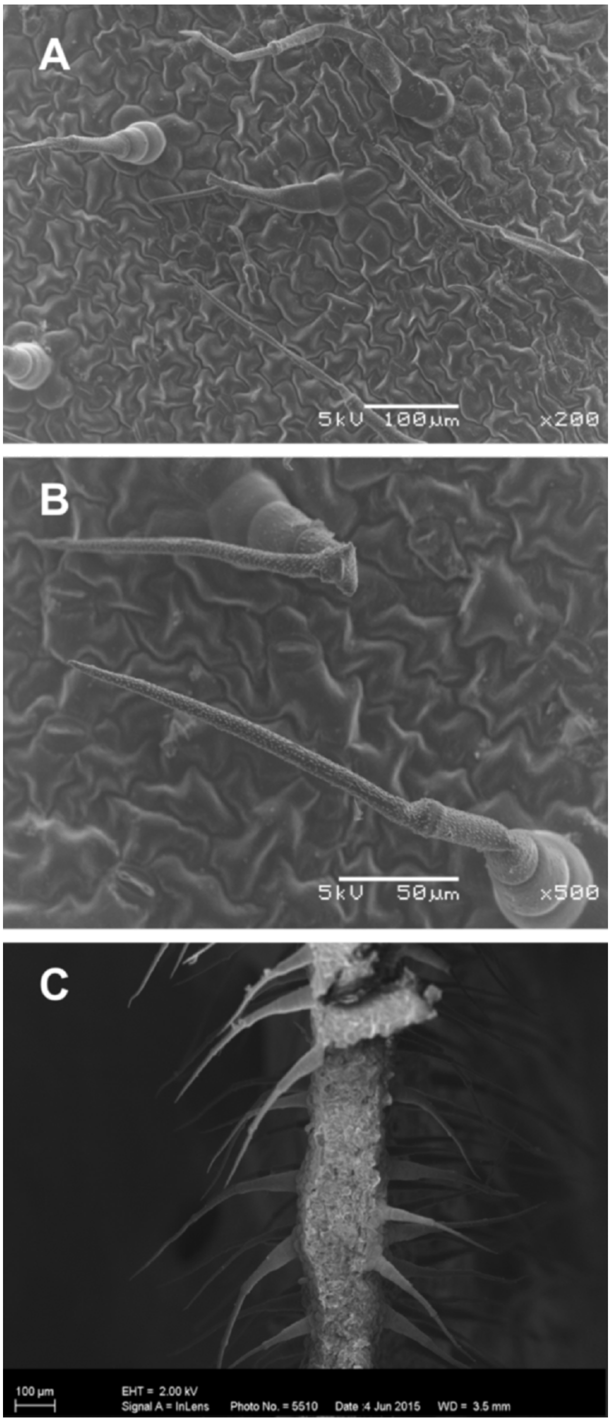

Figure S3. Scanning electron micrographs of *C. bonariensis* trichomes on leaf surface (A and B) and on cross section of leaf (C) showing trichomes on both adaxial and abaxial sides. Trichome cell walls with micropapillate sculpturing (B). The scale bar indicates 100 µm in image ‘A’, 50 µm in image ‘B’ and 100 µm in image ‘C’.

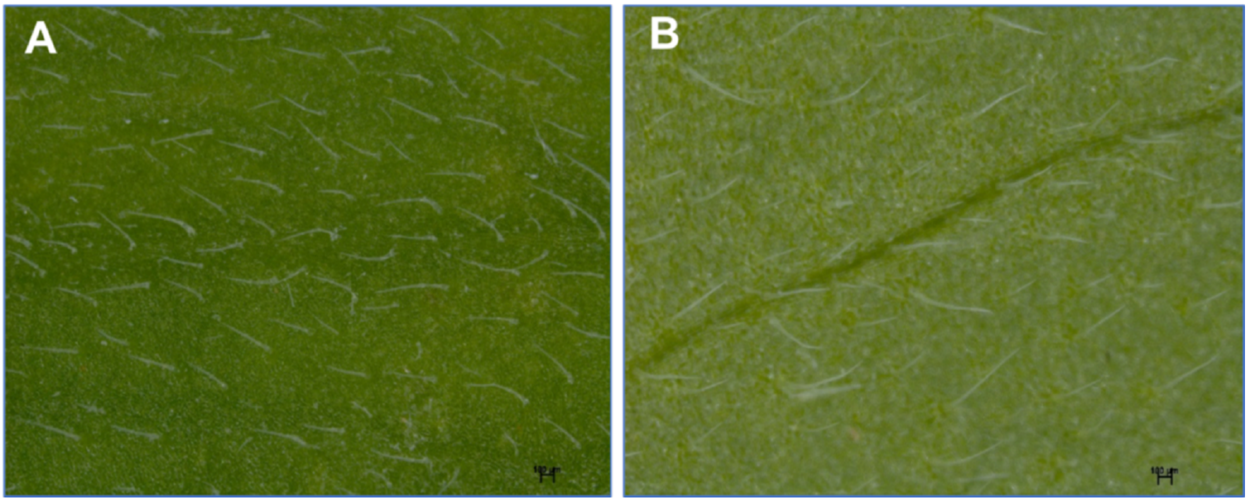

Figure S4. Light microscope images of *C. bonariensis* leaf surface showing numerous trichomes on the adaxial (‘A’) and fewer trichomes on the abaxial (‘B’) leaf surfaces. The scale bar indicates 100 µm.

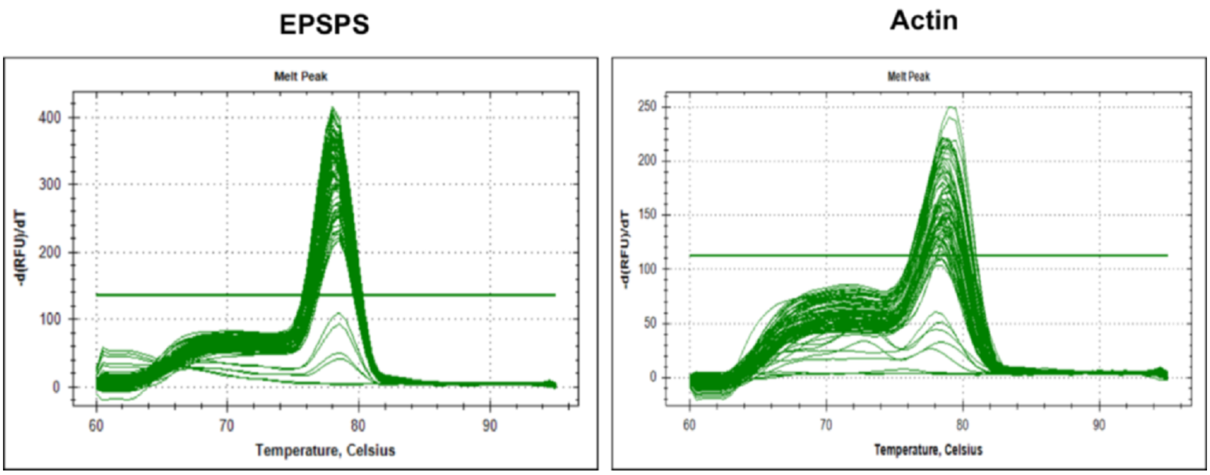

Figure S5. Melting curves for the *EPSPS* and *Actin* genes of *C. bonariensis.*

Supplementary table

Table S1. qRT-PCR primer sets used in the study

**Gene**	**Primer sequence**	**Amplicon Size** (bp)	**Efficiency** (%)
EPSPS	F 5’ – AAGGTTGCGGGACAAGCA – 3’<br>R 5’ – GACAGAGTTCTCTGTCCAGGTT – 3’	91	98.98
Actin	F 5’ - GTTCTCAGTGGTGGCTCAAC – 3’<br>R 5’ - TGATCTTCATGCTGCTGGGA – 3’	91	102.97
